# Vector-borne pathogen surveillance in a metagenomic world

**DOI:** 10.1371/journal.pntd.0011943

**Published:** 2024-02-22

**Authors:** Koray Ergunay, Brian P. Bourke, Nicole Achee, Le Jiang, John Grieco, Yvonne-Marie Linton

**Affiliations:** 1 Walter Reed Biosystematics Unit (WRBU), Smithsonian Institution, Museum Support Center, Suitland, Maryland, United States of America; 2 One Health Branch, Walter Reed Army Institute of Research (WRAIR), Silver Spring, Maryland, United States of America; 3 Department of Entomology, Smithsonian Institution–National Museum of Natural History (NMNH), Washington, DC, United States of America; 4 Department of Medical Microbiology, Virology Unit, Faculty of Medicine, Hacettepe University, Ankara, Turkey; 5 Department of Biological Sciences, Eck Institute for Global Health, University of Notre Dame, Notre Dame, Indiana, United States of America; 6 Naval Medical Research Center (NMRC), Silver Spring, Maryland, United States of America; Universidad Nacional Autonoma de Mexico, MEXICO

## Introduction

From its inception to its widespread usage, nucleic acid–based assays have become the mainstay tool in infectious disease diagnostics and surveillance. Often reliant on polymerase chain reaction (PCR) or other methods of amplification, they have been adapted for quantitation, high-throughput testing, automation, isothermal, real-time, multiplex, or miniaturized target detection [1]. Nucleic acid sequencing has gone through a slower but steady evolution, from Sanger sequencing to massively parallel or next-generation sequencing (NGS), which has currently become the standard approach for producing microbial genome data [2]. Concomitantly, characterization of microbial genome diversity has been expanding at an unprecedented pace, revealing an abundance of novel microorganisms, and rapidly enhancing public repositories of microbial genomes [3]. This massive accumulation of genomic data necessitates regular inspection and updating of repositories and detection approaches for accurate discrimination of microbial species. Here, we aim to describe some of the current challenges in microbial nucleic acid detection, particularly from vector and environmental sources.

## Pathogens and endosymbionts—Confounding issues

Current information reveals that almost all tick-borne and tick-associated pathogenic bacteria have closely related endosymbiotic relatives coexisting in ticks, as exemplified for *Rickettsia*, *Francisella*, *Borrelia*, and *Coxiella* [4]. Additionally, a range of unclassified taxa include members of *Anaplasma* and *Ehrlichia* under *Anaplasmataceae*, and various protozoa under the genera *Babesia* and *Theileria*. Against the backdrop of abundant and expanding genomic data, it becomes increasingly challenging to develop assays for species-specific detection and to accurately assess primer specificity for targets in silico. A well-documented example of these difficulties exists for *Rickettsia* species within the Spotted Fever Group, where a single genomic target is not sufficient to reliably discriminate species, but rather multiple targets, including 16S/23S ribosomal DNA, *ompA/B*, *gltA*, or *sca4*, are required [5]. Similarly, many purportedly species-level primers for *Francisella*, *Borrelia*, and *Coxiella* are likely to amplify not only the target species but also related endosymbionts or isolates of unknown or null pathogenicity in ticks. It is also being recognized that endosymbiont sequences may be integrated into host genome assemblies, the origin of which are still undetermined [6]. Thus, the difficulties in resolving pathogens from endosymbionts, microbes of null and nonpathogenicity, and even host/vector reference genomes available from public repositories pose significant challenges and confound efforts for microbial detection. Recognizing such challenges, it is therefore preferable to continuously review and improve primer and probe sequences as appropriate, being mindful of possible updates in nomenclature and official vector-borne pathogen taxonomy. Moreover, optimization of newly developed assays should incorporate spiking of appropriate matrices, such as pooled arthropods in addition to culture-derived isolates, for a realistic simulation of naturally occurring microbial diversity in field-collected samples. Information on commercially available assays should therefore also be reviewed and assessed accordingly.

Virus nucleic acid detection and identification present a range of difficulties. Many novel viruses, some closely related to the known pathogens, have been described in the last decade [3]. Given the widespread availability of nontargeted screening approaches, the number of newly described viruses is likely to expand and result in alterations to virus taxonomy. There are further information gaps regarding the epidemiology, host range, and public health impact of the viruses confirmed as human pathogens. Therefore, nucleic acid–based testing for such viruses will also require regular review to account for emerging local or global viral diversity.

Major arthropod vectors of human diseases, including mosquitoes and ticks, may be naturally infected with viruses related to pathogens. Mosquito-specific flaviviruses and tick-associated *Bunyavirales* are among the striking examples [7,8]. Sequences of these viruses might interfere with nucleic acid testing for pathogens, depending on the primer/probe design and virus genome region targeted for detection. Evidence for host genome integration of particular nonretroviral RNA viruses has also been documented in vector mosquitoes, ticks, and sandflies [9]. Recently, integrated virus replicase sequences from the tick-borne human pathogen Jingmen tick virus have been identified in ticks, with implications for nucleic acid screening targeting this region [10].

## Metagenome-based detection—Opportunities and challenges

Today, NGS technologies are widely available and implemented in most biological or medical processes that require sequence generation. For microbial detection, NGS can be utilized to sequence PCR amplicons, arbitrarily enriched samples, or, in a truly metagenome- or transcriptome-based fashion, all nucleic acids present in the sample [2]. The latter approach leverages the capability of detecting known pathogens as well as extremely divergent or novel microbial sequences without the need for prior information. Existing raw data sets accessioned in public repositories can be retrospectively screened for more recently described pathogens or novel microorganisms using updated reference databases and bioinformatic tools. It is possible to produce metagenome-assembled genomes (MAGs) for pathogens or nonpathogenic microorganisms by utilizing previously generated raw metagenome data. Despite these advantages, NGS technologies have important shortcomings that significantly affect robust microbial detection and identification.

One of the main challenges in metagenome-based approaches is the vast underrepresentation of microbial sequences, especially in vector arthropods and environmental samples. This might significantly jeopardize the detection of microbial sequences that mostly constitutes the needle in the haystack of host genomes and contaminants from the environment. Several enrichment approaches have been developed and utilized for specific microbial targets, such as viruses [11]. Furthermore, different commercially available NGS platforms may vary in their capacity to detect and characterize microbial genomes. Short-read sequencing (widely represented by Illumina) commonly incorporates a presequencing amplification-based enrichment step that might introduce bias for the assessment of microbial communities or diversity. Moreover, due to the production of uniformly short reads, a considerable sequencing depth and coverage must be maintained for reliable microbial taxonomic identification. Long-read sequencing (widely represented by Oxford Nanopore Technologies and Pacific Biosciences) typically require fewer reads for genome-wide coverage of microbial targets. However, both approaches ultimately rely on discriminating sections in the microbial genome (such as particular genes) to be captured in the output data for accurate species determination, particularly for bacterial or eukaryotic pathogens. In typical experimental settings for each platform, problems in discriminating pathogenic species of *Rickettsia*, *Francisella*, and *Coxiella* in ticks and *Plasmodium* in mosquitoes, in pooled or individual samples, are commonly observed. This can be partially overcome by increasing sequence depth by barcode multiplexing a lower number of samples per sequencing run, with the expense of increased financial burden.

Computational analysis of the NGS data constitute another bottleneck for the metagenome-based microbial detection. Initially, the raw output of any NGS run requires a series of software analyses for trimming, filtering, and quality control [12]. Subsequently, the sequences (or reads) undergo a taxonomic classification step, whereby each sequence is assigned to its respective taxonomic group by querying against reference databases. This can be accomplished by several approaches and software relying on similarity or composition, alignment or index-based tools, and using protein or nucleotide databases. For microbial identification, each approach has limitations that should be considered by end users. For example, effective species discrimination of closely related bacterial species would require different approaches than identifying extremely divergent or novel viruses. Microbial genome size, coverage, and overall representation all influence the accuracy of taxonomic assignment. While the initial processing and taxonomic classification of microbes require considerable computational resources and bioinformatics expertise, several web-based resources are becoming available for online data processing, some of which produce pathogen-tagged outputs [13]. Nevertheless, outputs from read taxonomic classifiers frequently require additional confirmation and assembly of consensus sequences for downstream analyses in silico. Establishing the identity of the detected microorganism and assessment of public health consequences require additional expertise.

## Biological relevance—A cautionary note

When used optimally, the capabilities of currently available targeted or metagenome-based methods enable detection and classification of minute amounts of genomes from a diverse spectrum of microbial species. However, the biological significance or relevance of any detection, and its translation into usable information for public health purposes, should be carefully considered, especially in vector and environmental samples [14]. Reporting of pathogens should adopt more stringent requirements, standards, and controls. The extent to which microbial detection correlates with transmission or emergence risks should be interpreted individually, considering all potentially contributing factors including vector ecology and disease epidemiology. Findings due to endogenous microbes and environmental contaminants should be carefully interpreted in each sample. Many novel viruses are represented only by genome sequences or MAGs and often lack in vivo isolates and information from well-characterized experimental infections [15]. Hence, causative associations with disease may require additional layers of evidence for pathogenicity. Moreover, findings from different assays might not be directly comparable. For example, a short segment of a microbial genome targeted by PCR may be absent from a metagenome dataset. Thresholds for detection and reliable species demarcation also vary significantly among assays.

## Concluding remarks

Metagenomic sequencing allows us to better identify pathogen emergence and describe microbial diversity in vectors or in the environment. However, the implementation of the available technologies is impacted by requirements of infrastructure, resources, and user expertise. Therefore, experimental and analytical approaches should be carefully preplanned to generate the information required for particular surveillance goals within the One Health concept, considering the interactions between human, animal, and environmental contributors.
